# Dataset on FAP-induced emergence of spontaneous metastases and on the preparation of activatable FAP-targeting immunoliposomes to detect the metastases

**DOI:** 10.1016/j.dib.2016.08.058

**Published:** 2016-09-03

**Authors:** Felista L. Tansi, Ronny Rüger, Claudia Böhm, Roland E. Kontermann, Ulf K. Teichgraeber, Alfred Fahr, Ingrid Hilger

**Affiliations:** a Department of Experimental Radiology, Institute of Diagnostic and Interventional Radiology, Jena University Hospital - Friedrich Schiller University Jena, Erlanger Allee 101, 07747 Jena, Germany; b Department of Pharmaceutical Technology, Friedrich-Schiller-University Jena, Lessingstrasse 8, 07743 Jena, Germany; c Institute of Cell Biology and Immunology, University Stuttgart, Allmandring 31, 70569 Stuttgart, Germany

## Abstract

The underlying data demonstrates that fibroblast activation protein (FAP) paves the way for fibrosarcoma cells, which require the proteolysis of the extracellular matrix (ECM) and basement membranes to intravasate from implanted subcutaneous primary tumors into blood vessels, be transported to distant organs where they extravasate from the blood vessels, reattach and proliferate to metastases. The data additionally shows that FAP, when overexpressed on fibrosarcoma cells induces their invasion and formation of spontaneous metastases in multiple organs, particularly after subcutaneous co-implantation of the FAP-expressing and wildtype fibrosarcoma. The raw and processed data presented herein is related to a research article entitled “Potential of activatable FAP-targeting immunoliposomes in intraoperative imaging of spontaneous metastases” (F.L. Tansi, R. Rüger, C. Böhm, R.E. Kontermann, U.K. Teichgraeber, A. Fahr, I. Hilger, 2016) [1]. Furthermore, evidence for the detection of FAP-expressing tumor cells and cells of the tumor stroma by activatable FAP-targeting liposomes is presented in this dataset.

**Specifications Table**

**Table:** 

Subject area	Biology, Chemistry
More specific subject area	Cancer biology and drug delivery systems
Type of data	Text file, table, figure
How data was acquired	Histology, Microscopy
Data format	Raw and analyzed
Experimental factors	Mice implanted subcutaneously with wild type and FAP expressing fibrosarcoma and used in Tansi et al. [1] were evaluated for metastases.
Experimental features	Histological evaluation of spontaneous metastases in mice with FAP expressing primary tumors.
Data source location	Jena, Germany
Data accessibility	Data is available with this article

**Value of the data**

• Identifying and targeting the molecular factors that influence cancer metastases is useful for the management of cancer.
• Overexpression of fibroblast activation protein (FAP) by fibrosarcoma cells influences their motility and metastasis formation potential and is exploited in the design of models of spontaneous metastases.
• Liposome-based fluorescent agents are valuable tools for monitoring of spontaneous metastases and image guided drug delivery and their preparation is valuable for research and drug discovery.

## 1 Data

The data presented herein comprises tabulated observations and tumor volumes of mice co-implanted with wildtype and FAP-expressing fibrosarcoma cells, as well as histological sections of the excised tumors and lungs. Also, a detailed data on the preparation of FAP-targeting immunoliposomes is presented. Images of tumor sections demonstrating the localization of the liposomal fluorescence in FAP-expressing tumor cells and myofibroblasts are also presented.

## 2 Experimental design, materials and methods

### 2.1 Design and analysis of a model of FAP-induced spontaneous metastasis

The wild type human fibrosarcoma cell line HT1080 and its stable FAP-expressing counterpart, HT1080-FAP were co-implanted subcutaneously in immune deficient nude mice [1] and the mice observed and evaluated for the emergence of spontaneous metastases.

#### 2.1.1 Determination of primary tumor size and presence of FAP-induced spontaneous metastases

The tumors were measured with a caliper and the tumor volumes were derived according to the literature [2]. The wildtype HT1080 cells produced xenografts with normal growth in 75% (15 of 20) and small tumors in 25% (5 of 20) of the animals, whereas the transfected HT1080-FAP cells revealed no or only very small xenografts in all the animals investigated (Table 1).

#### 2.1.2 Histological analysis and microscopic detection of spontaneous lung metastases and infiltrating cells originating from subcutaneous primary tumors

**Fig. 1:**
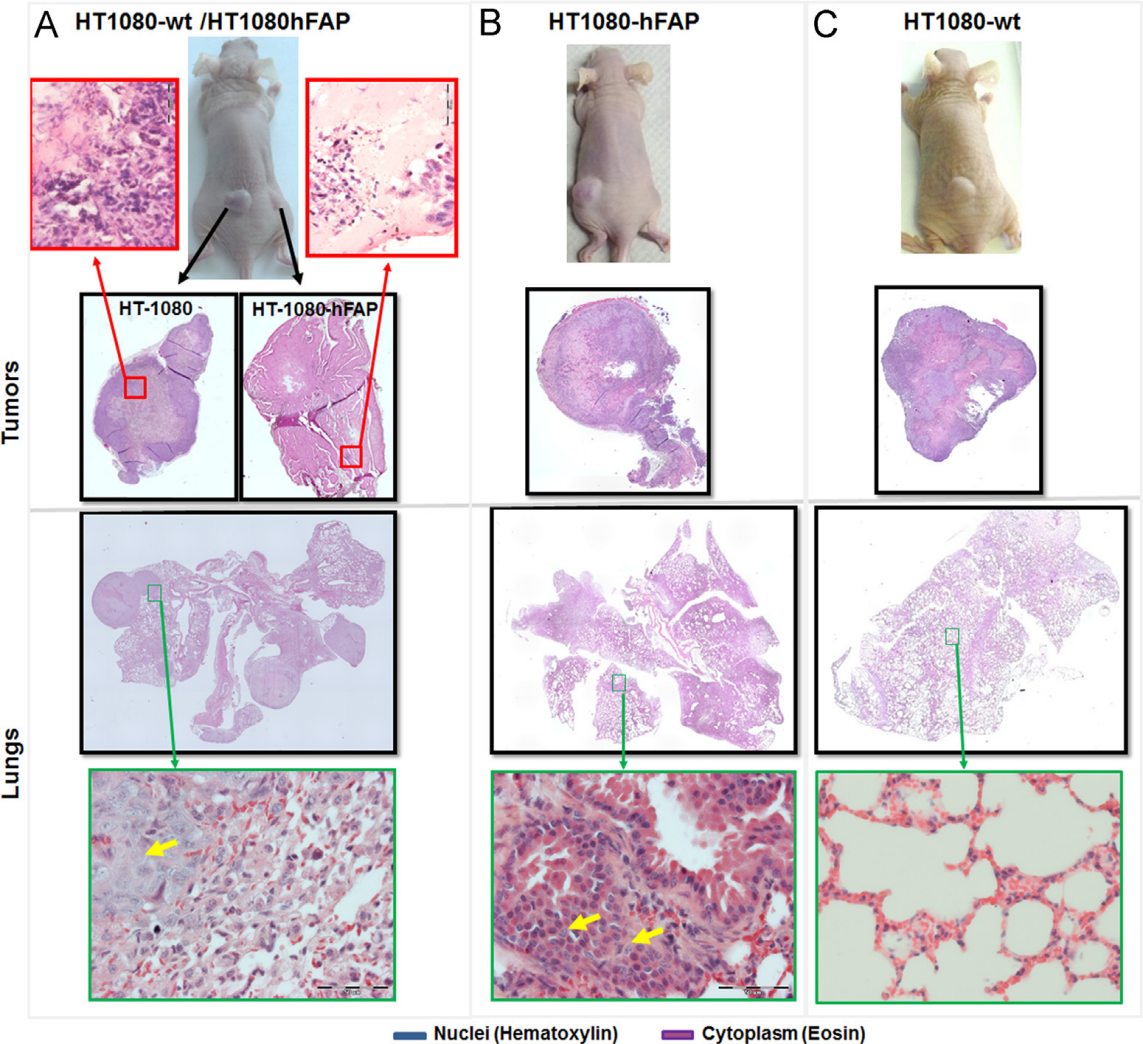
FAP induced motility of tumor cells and formation of spontaneous metastases in mice models. (A) Representative mouse implanted with both HT1080-hFAP and HT1080-wt, (B) with HT1080-hFAP alone and (C) with HT1080-wt alone and histological slices of the respective tumors and lungs excised 6–8 weeks post implantation. Histological stain of tumor and lung slices was with hematoxylin (nuclei) and eosin (cytoplasm). Whole mount microscopic images of the lung slice was done with the Keyence-BZ9000 microscope at a 4x magnification and the images fused. The detailed images were done at 60x magnification with an Olympus BX50 microscope. Yellow arrows point at infiltrating tumor cells in the lungs. Scale bars: 50 µm.

Histological slices of HT1080-hFAP and HT1080-wt tumors and lungs excised from mice models bearing single or double-implants were stained with hematoxylin and Eosin and subjected to microscopy. HT1080-hFAP tumors from mice with double implants revealed very few clusters of cells and areas of single cells migrating through the matrigel used for implantation, whereas slices of the HT1080-wt derived tumors revealed normal growth and distribution of the cells (Fig. 1, *Tumors*). Furthermore, histological analysis of the lungs from the animals bearing both tumors revealed a high level of lung metastasis and hyperplasia resulting from infiltrating tumor cells (Fig. 1A, *Lungs*). Opposed to this, mice implanted with the FAP expressing HT1080-hFAP tumors alone showed slow but relatively improved tumor growth (Fig. 1B, *Tumors*) and a high level of lung hyperplasia of infiltrating tumor cells, but no visible large lung metastases (Fig. 1B, *Lungs*). On the other hand, mice implanted with the wildtype HT1080 alone revealed rapid tumor growth, and the respective lung slices showed no significant abnormalities (Fig. 1C).

### 2.2 Preparation of activatable liposomes

#### 2.2.1 Materials

The egg phosphatidylcholine (EPC) was purchased from Lipoid (Ludwigshafen, Germany), whereas 1,2-distearoyl-sn-glycero-3-phosphoethanolamine-N-[methoxy (polyethylene glycol)−2000] (ammonium salt) (mPEG_2000_-DSPE), 1,2-distearoyl-sn-glycero-3-phosphoethanolamine-N-[maleimide(polyethylene glycol)−2000] (ammonium salt) (Mal-PEG_2000_-DSPE) and 1,2-dioleoyl-sn-glycero-3-phosphoethanolamine-N-(7-nitro-2-1,3-benzoxadiazol-4-yl) (ammonium salt) (NBD-DOPE) were from Avanti Polar Lipids (Alabaster, USA). Cholesterol, Tris(hydroxymethyl)-aminomethane (Tris) and 4-(1,1,3,3-Tetramethylbutyl)phenyl-polyethylene glycol (Triton-X100) were from Sigma (Taufkirchen, Germany). The near infrared fluorescent dye, DY-676-COOH was purchased from DYOMICS GmbH (Jena, Germany).

The recombinant FAP specific single chain antibody fragment (FAP-scFv) containing a c-terminal cysteine residue was purified from transformed *Escherichia coli* as reported earlier [3].

#### 2.2.2 Preparation of quenched DY-676-COOH containing liposomes (LipQ)

The liposomes were prepared by the standard film hydration and extrusion method composed of the lipid combination EPC:Chol:mPEG_2000_-DSPE at a molar ratio of 6.5:3:0.5 as reported in detail elsewhere [4]. The resulting DY-676-COOH containing liposomes were dispersed in buffer (10 mM Tris, pH 7.4) to have a final lipid concentration of 50 mM.

#### 2.2.3 Conjugation of FAP׳scFv fragments to get FAP-targeting immunoliposomes

FAP-Immunoliposomes (FAP-IL) were obtained by post-insertion of scFv’FAP-Mal-PEG_2000_-DSPE micelles to preformed quenched liposomes as described earlier [5]. For this purpose, the Mal-PEG_2000_-DSPE micelles were prepared and conjugated to reduced ligands (scFv’FAP) according to Mack et al. [6]. The resulting immunoliposomes were also dispersed in buffer (10 mM Tris, pH 7.4) to have a final lipid concentration of 50 mM.

#### 2.2.4 Quantitation of liposomal encapsulated DY-676-COOH

To determine the amount of DY-676-COOH encapsulated, a calibration curve was prepared with different concentrations of the free DY-676-COOH (0, 82, 124, 247, 494, 741, 988 nM) in 10 mM Tris buffer, pH 7.4 containing 0.1% Triton X100. The liposome samples were incubated for 5 min at RT in buffer containing 1% Triton X100 and afterwards diluted with 10 mM Tris buffer, pH 7.4 to a final Triton-X100 concentration of 0.1%. All samples were measured in triplicates on a Fluostar Optima microplate reader (BMG Labtech, GmbH, Ortenberg, Germany) at *λ*=700 nm after excitation at *λ*=645 nm.

### 2.3 Detection of tumor stromal fibroblasts with FAP-targeted immunoliposomes (FAP-IL)

**Fig. 2:**
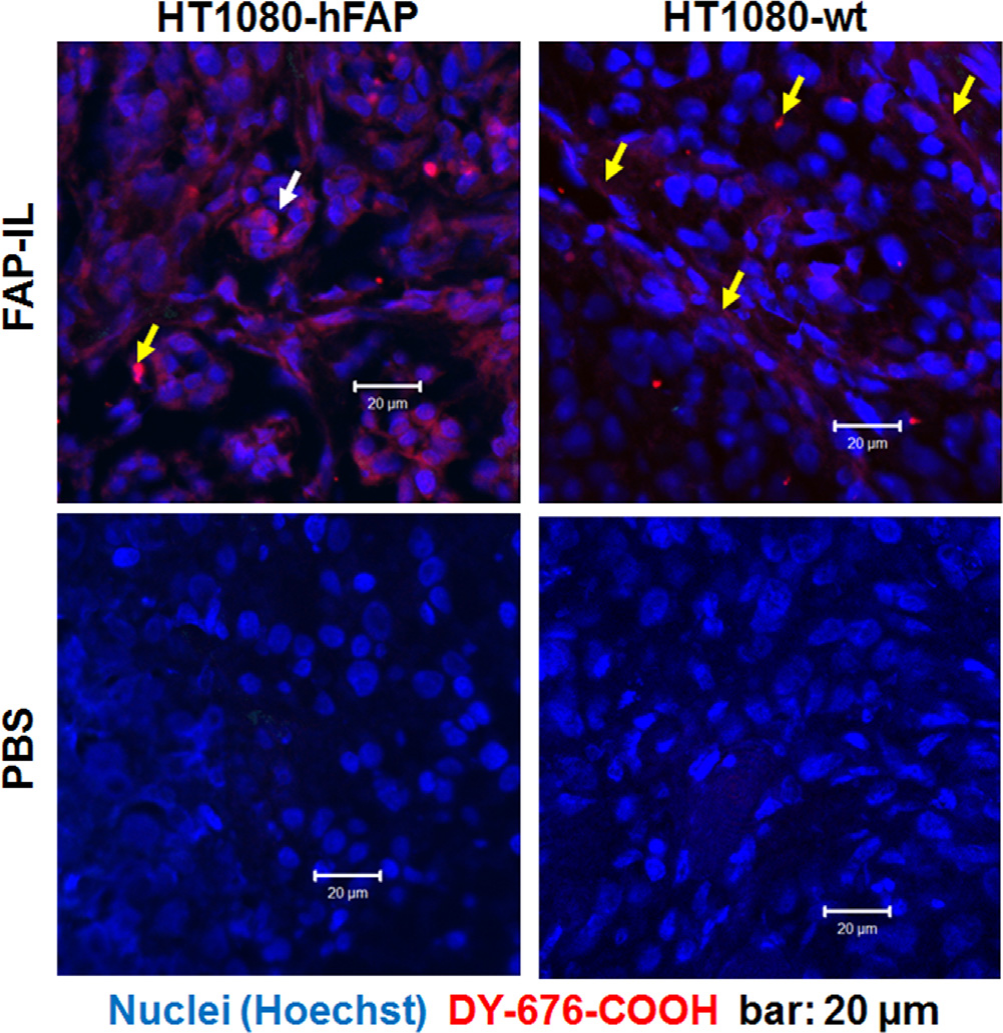
Microscopic detection of FAP-IL based fluorescence in tumor slices. Histological slices of FAP-expressing (HT1080-hFAP) and wild type FAP-negative (HT1080-wt) tumor models reveal characteristic FAP-IL fluorescence in tumor cells (white arrow) and tumor stromal cells (yellow arrows) 24 h post intravenous injection. No Fluorescence is seen after injection of buffer (PBS).

Subcutaneous tumors were excised from mice 24 h post intravenous injection of FAP-IL (30 µmol/kg body weight) or PBS. Slices of the paraffin embedded tumors were prepared, dewaxed as described in [1] and subjected to confocal laser scanning microscopy. The data demonstrates FAP-IL-based fluorescence in tumor cells and stroma of the FAP-expressing tumor model and only in the stroma of the wild type tumor model (Fig. 2, *FAP-IL*). No fluorescence is seen in tumors from control mice which were injected with PBS (Fig. 2, *PBS*).

## Figures and Tables

**Table 1:** Tumor volumes of subcutaneous HT1080 and HT1080-FAP tumors 6 weeks or 8 weeks (grey shadow) post implantation and respective observations made with respect to metastases.

hp: hyperplasia, met: metastatic lesion.
