# Supplementary material for: Dataset on FAP-induced emergence of spontaneous metastases and on the preparation of activatable FAP-targeting immunoliposomes to detect the metastases
Source: Data Brief. 2016 Sep 3;9:143–8. doi: 10.1016/j.dib.2016.08.058 (PMC5018079; doi:10.1016/j.dib.2016.08.058)
Supplement: Supplementary file 1 — Supplementary material [file mmc1.docx]

**Design of a model of spontaneous metastases and preparation of activatable FAP-targeting immunoliposomes to detect the metastases**

Felista L. Tansi^1*^, Ronny Rüger^2^, Claudia Böhm^1^, Roland E. Kontermann^3^, Ulf K. Teichgraeber^1^, Alfred Fahr^2^ and Ingrid Hilger^1^

^1^Dept. of Experimental Radiology, Institute of Diagnostic and Interventional Radiology, Jena University Hospital - Friedrich Schiller University Jena, Erlanger Allee 101, 07747 Jena, Germany.

^2^Department of Pharmaceutical Technology, Friedrich-Schiller-University Jena, Lessingstrasse 8, 07743 Jena, Germany

^3^Institute of Cell Biology and Immunology, University Stuttgart, Allmandring 31, 70569 Stuttgart, Germany

***Contact email:** Felista L. Tansi: felista.tansi@med.uni-jena.de

#

# Conflict of Interest

The authors declare no conflict of interest.
